# Foot health education for people with rheumatoid arthritis — some patient perspectives

**DOI:** 10.1186/1757-1146-5-23

**Published:** 2012-08-31

**Authors:** Andrea S Graham, Alison Hammond, Steven Walmsley, Anita E Williams

**Affiliations:** 1 Centre for Health, Sport and Rehabilitation Research, University of Salford, Frederick Road, Salford, UK; 2 Directorate of Prosthetics, Orthotics and Podiatry, University of Salford, Frederick Road, Salford, UK

**Keywords:** Podiatry, Patient, Foot health education, Rheumatoid arthritis

## Abstract

**Background:**

Patient education is an important component of foot health management for people with rheumatoid arthritis (RA). The content and strategies for delivery require investigation in relation to the patients’ needs. This study explores patients’ experiences of foot health education, to inform how the patients’ needs could be identified in clinical practice and inform effective education delivery.

**Method:**

A focus group was used to collect data. The dialogue was recorded digitally, transcribed verbatim and analysed using a structured thematic approach. Member checking and peer review added to credibility of the data.

Six themes emerged; (i) content and purpose of patient education – what it should be, (ii) content of patient education – what it should not be, (iii) timing of information on foot health, (iv) method of delivery, (v) ability to engage with foot health education and (vi) the patient/practitioner relationship.

**Conclusions:**

This study identified aspects of patient education considered important by this group of patients in relation to content, timing and delivery, forming the basis for further research on clinical and patient focussed outcomes of patient education.

Identifying health education needs and provision of supportive verbal and written information can foster an effective therapeutic relationship, supporting effective foot health education for people with RA.

## Background

National Health Service reviews and reports focus on the need for increased self-management in the overall management of patients with long-term conditions, such as Rheumatoid Arthritis (RA) [1,2]. In support of this the Department of Health ‘information revolution’ [1] provides resources that aim to improve health related behaviour, support aspects of self-management and thereby maximise the potential for health benefits [1]. For people with RA, it is known that patient education, including verbal and written information, self-study, websites and psycho-educational programmes, have a positive effect in relation to disease management and general health [3].

Patient education is recognised as important for people with RA in relation to foot health [4-6]. Up to 80% of people with RA report foot pain on a regular basis [7,8]. Providing education during podiatry consultations, in the form of information on the purpose and use of clinical interventions, such as foot orthoses and specialist footwear [9], could potentially improve patients’ use of them [10].

The skills required to deliver patient education, are now embedded in the undergraduate curriculum and are considered a core component of podiatry care. Podiatrists perceive it as a valued and beneficial activity supporting aspects of foot management that patients can perform themselves [11]. Despite recommendations for an increased role of the patient in foot health [4-6], little is known from the patient perspective.

It is important to consider that practitioners and patients may have diverging opinions about what is important [12]. Despite benchmark standards [13] that state that patient education should be patient centred, based around patient need, there is some evidence that this is not being fully met [14,15].

For foot health education to meet the needs of the patient and support self-management we need to understand their perceptions of what and how it is currently delivered. Therefore, the aim of this study was to explore patients’ experiences of foot health education, in order to inform how the patients’ needs could be identified in clinical practice. In achieving this, effective education as an intervention could be delivered.

## Methods

### Design

A qualitative approach using focus groups was selected because: focus groups are an effective method of exploring people’s experiences of their health condition and its management; they produce a richness of data from a small group of people simultaneously [16]; and can generate data where there is little existing knowledge [17]. Four to nine participants were required for the focus group. This is considered to be the optimum size for such interviews [16,18,19] and appropriate for the generation of data for analysis using a thematic framework [20].

### Participants

Using a purposive sample framework, six people aged over 18 years, with a diagnosis of RA [21] and foot problems; able to read and speak English; and able to provide written consent were recruited from a North West England rheumatoid arthritis support group. People with severe mental illness were excluded due to their inability to fully consent.

### Procedures

Following ethical approval from the University of Salford, the Chair of the RA support group distributed an invitation letter, participant information leaflet and response form to all members with RA. The information leaflet provided contact details of the first author (AG) to allow members interested in participating the opportunity to ask questions about the study. Immediately prior to the focus group a presentation about the study was given to the RA support group. Members then had the additional opportunity to ask questions and written consent was obtained from those members who wished to participate.

Trigger questions with additional prompts were created by the first author (AG) and agreed by the co-authors. The questions were based on the first author’s previous knowledge of foot health education provision to people with RA and focus group work with practitioners. Potential participants were invited to view the trigger questions before consenting to take part in the focus group. This ensured that the questions could be clearly understood and took into account the views of the participants as collaborators in the research process [22]. No amendments were required. The focus group took place where the participants met as a support group, providing a familiar and private environment [23]. The members were advised that the focus group would last approximately one hour, using the interview questions to generate discussion. Breaks could be taken at any time, if required. The focus group was facilitated by AG and an independent observer, (SW) made additional observations and took field notes. The first author transcribed the dialogue verbatim.

Focus group questions: details of the questions used to generate participant discussion during the course of the patients’ focus group

**In your opinion, what is Patient Education**? (in relation to foot health)

What do you think the purpose of it is?

What is the usefulness of it?

What kind of information is given?

Prompts:

What kind of things are you told about Rheumatoid Arthritis?

About Podiatry?

About what can be done for your feet?

When is patient education given?

Prompts:

Think about when you were first diagnosed/first saw a podiatrist – were you given any foot health related information or advice then?

Have you been given any information/education about your feet since then, if so when?

Is this something you discuss regularly or was it a ‘one-off’?

How is the information/education provided for you?

Prompts:

For example were you simply given verbal advice?

Did you receive any written information such as leaflets provided by the Trust, AR UK, NRAS, from the podiatrist or any other Healthcare professional relating to your feet?

Were you prompted to use any websites?

What did you think about the resources that you were provided with?

In your opinion, what prevents you from obtaining the foot health information/education that you want?

Prompts:

Is there anything that stops you from getting the information or advice that you need at the time that you need it?

How easy is it for you to access your podiatrist for example?

Do you know where to go for the right kind of information?

Do you have easy access to the internet for example?

### Data analysis

The participants verified the transcription, which was sent electronically to the chair of the group for dissemination, to support the trustworthiness of the data [24,25]. Paper copies of the transcription were available on request. The verified dialogue transcription was subject to thematic analysis [20]. A thematic framework was used, allowing the researcher to illustrate the main themes within the text and make transparent the methodical systematisation of textual data. To achieve this, a six-stage process was used involving: coding the text; theme identification; thematic network construction; description and exploration of networks; summarisation of networks; and pattern interpretation [26]. The data was categorised into ‘Basic’ and ‘Organising’ themes (Table 1). This approach acknowledges the researchers’ experience and knowledge of the subject being researched and the influence of this throughout the data collection and interpretation. The thematic analysis framework was agreed by one of the co-authors (AW) to evaluate validity of the data and exemplars were extracted to demonstrate truthfulness of the data within each theme [24,25]. 

**Table 1:** Outline of the basic and organising themes developed from the thematic analysis

**Basic Themes**	**Organising Themes**
· Information Provision	The Content and purpose of Patient Education – what it should be.
· Signposting	
· Preparedness	
· Explanation of service and interventions	
· Self-management	
· The podiatrists role and scope of practice	
· The role of other Allied Health Professionals	
· Information from internet sources	The Content of Patient Education – what it shouldn’t be.
· Fear of the future – prognosis for foot health	
· Comparison of foot health in RA to that in other diseases	
· Fear of interventions	
· Timing of referral to podiatry	Timing of Information on Foot Health
· Timing of delivery of educational material	
· Time available within a consultation	
· Time to reflect	
· Internet resources	Method of delivery
· Group Education	
· One-to-one	
· Written	
· Verbal	
· Finance	Ability to engage with Patient Education
· Time	
· Access	
· Information Retention	
· Helpfulness	The Patient - Practitioner Relationship
· Being listened to	
· Influence of gender	

## Results

Out of twenty members of the support group approached, six participants who met the inclusion criteria initially consented. One was unable to attend the focus group due to ill health. All five participants were women, with a mean age of 62 years (SD 5.3) and mean disease duration 5.9 years (SD 2.7). All participants had experienced foot problems and had received National Health Service (NHS) podiatry services. Two participants had attended group Patient Education sessions, relating to RA but not foot health, subsequent to their diagnosis. The remaining participants had not received any formalised patient education. Participants’ names have been replaced with a pseudonym for confidentiality.

### Global theme: Barriers to engagement with foot health education

The unifying global theme was that there are barriers to receiving foot health education from podiatrists, leading to information being sought from sources that resulted in confusion and fear. In support of this six organising themes emerged:

### The content and purpose of patient education – what it should be

Participants considered that patient education (PE) was primarily an information resource that could guide them to other sources of information, such as the Internet. As Mary highlighted:

"“(patient education is) what you are told by your Specialist, what you can find out on the web and other sources.”"

Identifying how to access foot health information resources and what they should know about foot health were issues for all the group:

"“A lot of it is that you don’t know what you don’t know!” (Kitty)."

All participants considered they had received little or no information regarding their foot health. However, they wanted to be prepared for what might lie ahead: potential foot-related morbidity; prevention of foot health deterioration; and the side effects of medication on foot health. They also wanted information on the availability of foot health services and foot health interventions:

"“You need details of specific foot problems… the sort of thing we all experience really, like fallen arches or pains in your toes, what this is caused by or how you can help it (and) what treatment is available for each problem” (Mary)."

Participants wanted information to facilitate safe and effective self-management. Some had received general footwear advice. However, they expressed disillusionment with it, as their individual needs had not been considered, such as their ability to find accommodating footwear:

"“She said wear trainers, I can’t even wear trainers…my instep is so high” (Joan)."

None of the participants had been informed about the scope of practice of podiatry but did perceive that ‘chiropodists’ and ‘podiatrists’ were different in relation to the level of expertise. This indicates confusion, as in reality they are the same:

"“You would expect them [podiatrist’s] to know more in depth about your foot problems really, a chiropodist I would look on as more for cosmetic things really like hard skin, toenails” (Mary)."

Participants emphasised the need for clarification on the podiatrists’ scope of practice, as well as that of other health professionals involved in foot health.

### The content of patient education ’ what it should not be

Most participants had accessed foot health information through the Internet and found it frightening and overwhelming, reinforcing their fear of developing foot problems:

"“Sometimes they cannot be very helpful, or they can tell you too much, they’ll blind you with science which you don’t understand or they’ll tell you something and you think ‘oh my feet are going to drop off!’” (Lynne)."

This negative view of their future foot health was further reinforced by comparing their foot pathology with those of others they knew (friends/family) with chronic diseases, such as diabetes:

"“…they get told that when they’ve got diabetes, that different things can happen to them [their feet]” (Bernice)."

Lack of appropriate education and information about interventions often invoked fear, anxiety and concern, particularly in relation to footwear styles required to accommodate both changing foot shape and orthoses:

"“It’s not possible to get something that works and is fashionable as well is it?” (Mary)."

Mary’s question highlights the participant’s concerns relating to the image that therapeutic footwear represents and the function that it provides and this is further reinforced by Lynne who stated that:

"“Well none of us [indicates to the group] has special shoes and if we did, I think we'd all throw them to the back of the wardrobe as soon as we got them home. Because I've seen them… and I’d have to be dead to wear them.” (Lynne)."

### Timing of information on foot health

Early referral to a podiatrist was considered crucial for timely access to appropriate foot health information. Participants stated that such information should be presented in a way that was not overwhelming. It should allow them to first absorb the meaning of being diagnosed with RA:

"“You need to have a bit [of information] to tell you what can happen to your feet when you’ve got RA, but more when you go to see the podiatrist because by then you’ll have soaked in a bit, you can take a bit more.” (Lynne)."

Limited time during consultations was perceived as preventing foot health questions being raised:

"“…and then there’s the time factor as well, if I go into this podiatrist and say ‘what are you going to do for me?’ there’s only so much time.” (Joan)."

Time for reflecting on information provided was deemed essential to enable asking further questions at subsequent appointments.

### Method of delivery

The Internet was the most accessed resource. Frustration with limited information about RA and feet was expressed. Certain websites were considered too difficult to navigate to find the right information:

"“I had a terrible time with the NHS website, never found what I want.” (Lynne)."

Group education, provided by a range of health professionals, was considered best for arthritis-related information and self-management strategies within a supportive environment. Participants also considered that group education could provide information relating to topics they had not thought about. However, two participants who had attended education groups found them of little benefit. They were frustrated that group leaders allowed more vociferous individuals to dominate:

"”I can remember going to a group and getting so exasperated with a guy that I ended up telling him I’d come to listen to the tutor, not him.” (Kitty)."

The majority of participants had experienced one-to-one ‘verbal’ foot health education about general foot health issues together with an explanation of interventions, such as foot orthoses. The effectiveness of the patient/practitioner relationship influenced both the information provided and whether the patient’s agenda was identified:

"“…I thought he’s not really picking up on the main reason why I’d actually gone to see him.” (Mary)."

None had received written foot health information from any health professional, including podiatrists. Leaflets were viewed as an extremely useful ‘aide memoir’ as they considered that RA affects retention of information. Leaflets were considered useful to impart general information, such as frequently asked questions and were a reference source about who to contact for attention to foot problems.

### Ability to engage with foot health education

The financial cost of improving foot health behaviours, such as buying appropriate footwear and aids to facilitate self-management, were seen as barriers to engaging with advice:

"“They (long-handled files) are expensive if you go to a mobility shop, which is the only place you’ll get them.“ (Lynne)."

Other education resources, such as local support groups, can incur costs, which could be a barrier to people joining. Further, they perceived that practitioners experienced many time pressures and subsequently felt unable to approach Podiatry services to receive education.

The participants unanimously voiced that there was a distinct lack of information, which provided explanation without inciting fear and anxiety:

"We didn’t have the insight to ask for the information before because we didn’t know there was any available… it’s not there, podiatry wise it’s just not there.” (Lynne)."

### The patient/practitioner relationship

Generally, when advice or foot-health education was provided, they considered podiatrists to be helpful. Despite this, they considered that their point of view was often not heard, being dismissed without their key concerns being addressed:

"“…I went back again and said I can’t wear these [insoles] except for in my boots and they said ‘oh well you’ll get used to them’ and sent me home. And that were it, that’s the amount of information I got.” (Lynne)."

Participants considered female practitioners had a greater understanding of their needs:

"“I got on better with a female one (podiatrist)…she was absolutely brilliant, I felt I got a lot out of the appointment, the orthotics seemed to work better and she did give me a lot of information.” (Mary)."

## Discussion

Using focus group methodology and a thematic approach to data analysis has revealed a richness of data about the participants’ experiences and opinions about the purpose, content, methods of delivery and barriers to foot health education provision.

The small sample size and restricted geographical area means that caution must be taken in generalising the results to the wider population. The homogenous nature of the focus group participants could have led to sample bias and may have influenced the results due to the gender of the group and group facilitator. As the participants were members of a patient support group, it could be argued that this was a group of highly motivated individuals who were well informed with regards their condition and the health system, influencing the results further. The influence of the ‘groupthink’ phenomena could be considered a limitation in the use of focus groups, especially where the group has a high degree of cohesiveness and homogeneity [27]. However, the aim of the focus group was to gain insight into patients’ views on foot health education and to this end the methodology was appropriate. The limitations and criticism of using established groups can be countered by the benefits that using participants from an already established support group can add richness to the data as the group are more aligned to the research topic [19].

The strengths of this study lie with congruency of the overall themes that emerged from this and a previous study that revealed the practitioners’ perceptions with regards to the purpose, timing, content, best methods of delivery and barriers to the provision of foot health education [28], allowing triangulation of the data sources to better understand the area. This agreement reinforces the need for the development of a foot health education strategy that embraces both perspectives [29]. Identifying service users’ views as part of the development of foot health education has already been shown to be successful in an elderly population [30].

The role of health professionals in foot health management, accessing foot health services, general foot health information in the context of RA and good foot care self-management practices were considered to be essential components of foot health education provision. These areas have also been identified as key topics within foot health guidelines [5].

These participants were confused about the role of ‘podiatrists’ and ‘chiropodists.’ This resulted in a lack of clarity about the services they could access and what to expect from them. Discarding the title ‘chiropodist’ may help to improve understanding of the podiatry profession from the public perspective, an issue that has been identified by members of the podiatry profession [28,31-33]. Where ‘specialist’ roles were discussed during the focus group, there was the perception that the term ‘specialist podiatrist’ generated more confidence in the practitioner. Information about the podiatrist’s role and scope of practice is required to ensure that patients are aware of the level of expertise they can expect from the individual practitioner.

When foot health information was sourced it was reported as “frightening” or written in language that was difficult to interpret. All participants had used the Internet to seek foot health information, suggesting it is a well utilized resource. There is no lack of web based foot health information. Arthritis Research UK and the National Rheumatoid Arthritis Society (NRAS) provide resources that address foot health from both a general and RA specific perspective [34,35]. However, the participants were unaware of these resources, although all were members of NRAS. Patients concerns about locating high quality, patient-centred information relating to RA have been identified [36]. The findings of this study support this. Furthermore, a study of podiatrists found they directed patients to these web sites infrequently [28] potentially reinforcing patients’ perception that there is a lack of such information.

Participants wanted patient education leaflets to support verbal information given during consultations. Written information was considered an aide memoir, prompting questions at future appointments, especially as there is a risk of being ‘overwhelmed’ with information at the point of diagnosis. Written information for people with RA is considered the most effective way for people to refer to information once they have left the hospital setting [37]. The participants in this study viewed that RA had a negative impact on memory retention with pain and depression leading to poor cognitive function [38-40]. Written information was thus seen as highly valuable. Despite evidence for the effectiveness of patient information leaflets being weak [41], they are beneficial in increasing patient knowledge in the short term [42] but this must be individualised and supported by the practitioner for it to be effective [43], although this evidence relates to people with diabetes, the same may be true in RA.

Generally, group education was not viewed as beneficial by those members who had attended these sessions, as neither foot health education nor self-management was addressed. The potential value of group education was thought to be in providing a supportive environment for general foot health information and self-management education, if planned and facilitated appropriately. The implementation of group foot health self-management programmes for people with RA may be an effective method of delivery, providing members can perform self care tasks, such as basic nail cutting [44].

During their consultations with health professionals, participants found that individual information and education was often not provided as limited consultation time restricted them from asking questions. Individuals without foot pathology or few symptoms may not request foot health information, as they perceive their needs to be minimal [45]. However, within the context of a patient-centred consultation it is still important to identify their educational needs early in the disease [5]. This view was strongly articulated by the participants, as they felt let down and un-prepared for the way in which RA affected their feet and thus their daily activities. The feet are often the first part of the body to be affected in RA [7] with most experiencing foot pain early in the disease [8]. It is therefore essential to provide foot health education in a timely and targeted way.

In this study, the development of a strong and trusting therapeutic relationship was viewed as a critically influential factor for appropriate education. McInnes et al. [46] advocate a timely and individualized approach to diabetes foot health education provision. This requires investment of the practitioner’s time and identifying the patient’s agenda through using motivational interviewing techniques [47]. Identifying a person’s ‘readiness’ to change and motivation to engage in positive health behaviours is a key component of a patient-centred approach and should be undertaken during the course of any consultation [48].

Participants described the experience of being ‘listened to’ more by female practitioners as resulting in positive outcomes. This perceived higher level of empathy was also identified in a study with practitioners, who found it easier to advise female patients on ‘difficult’ foot health issues such as foot wear styles [28]. ‘Gender related communication skills’, most notably ‘patient-centeredness’, as opposed to gender alone, are thought to influence the development of a positive therapeutic relationship [49]. Although female practitioners are more likely to exhibit such skills [50], this does not preclude male practitioners from developing and demonstrating them. Thorough assessment and developmental feedback in relation to communication skills at undergraduate level may ensure similarities in development by male and female practitioners. It should be taken into consideration that the participants and facilitators of this study and the study with practitioners [28] were all female. The fact that the group participants and facilitators were of the same gender could have influenced the results. The development of a dynamic discussion is more likely where there is group homogeneity from both a gender and shared experience perspective [51]. Further research, exploring the perspectives of men, could provide a more comprehensive picture of the foot health education needs of people with RA.

Patients in this study wanted access to information from a variety of sources, together with a tailored approach and verbal explanation, to meet their needs. Group education was considered beneficial if structured, with ground rules applied so that individual needs were respected. However, patients strongly considered information should be staged according to their needs and preferences as their disease progressed. To achieve this, the patients’ needs must be identified to guide them to the most appropriate foot health information. An Education Needs Assessment Tool, focusing on RA and its management has been developed and evaluated [52]. A similar approach to identifying foot health educational needs would enable practitioners to tailor their education provision to patients’ needs.

## Conclusions

This study provides insight into the patient perspective on foot health education provision for people with RA. There were clear similarities to practitioner perspectives [27]. The data will inform a survey to ascertain the views of a wider population of people with RA and Podiatrists.

Time is needed during consultations to ascertain patients’ needs and readiness to engage in positive foot health behaviour. Written information, supported with a practitioner’s explanation and tailoring to the patients’ needs, will reduce anxiety and facilitate better patient education and patient uptake of positive foot health behaviours. Further, this will encourage a therapeutic relationship enabling positive health behaviour and self-management, as recommended in the Darzi report [2]. Teaching and assessment of undergraduate communication skills to ensure patient-centred consultation skills may result in an improved patient experience of the consultation and reduce gender bias overall.

## Abbreviations

NHS: National Health Service; NRAS: National Rheumatoid Arthritis Society; RA: Rheumatoid Arthritis.

## Competing interests

The authors declare they have no competing interests.

## Authors’ contributions

AG conceived and executed the study design (with contributions from AW and AH), interpreted the findings with assistance from SW and drafted the manuscript with assistance from AW and AH. All authors read and approved the final manuscript.
